# Concordance Analysis of ALEX‐2, ImmunoCAP, and Prick Test for Respiratory Allergies in Lima, Peru

**DOI:** 10.1002/iid3.70325

**Published:** 2026-01-12

**Authors:** César A. Galván, Rafael Durán, José Ignacio Larco, Juan Carlos Gomez de la Torre

**Affiliations:** ^1^ Emedic Salud Lima Peru; ^2^ Clinical Allergy Service Clínica Internacional Lima Peru; ^3^ Human Nutrition and Food Research Group (GINAH) Universidad Científica del Sur Lima Peru; ^4^ Allergy Unit Clinica San Felipe Lima Peru; ^5^ Laboratorios ROE Lima Peru; ^6^ Instituto de Investigaciones en Ciencias Biomédicas Universidad Ricardo Palma Lima Peru

**Keywords:** asthma, immunoglobulin E, respiratory hypersensitivity, rhinitis, skin test

## Abstract

**Background:**

Accurate identification of respiratory allergen sensitization is essential for managing asthma and allergic rhinitis (AR). While Skin Prick Test (SPT) and ImmunoCAP are standard diagnostic tools, exploratory comparative data for the novel Allergy Explorer 2 (ALEX‐2) test in Latin American populations remain scarce. While Skin Prick Test (SPT) and ImmunoCAP are standard diagnostic tools with limited allergen panels per test, the novel Allergy Explorer 2 (ALEX‐2) multiplex platform can simultaneously assess multiple allergens. However, exploratory comparative data for ALEX‐2 in Latin American populations remain scarce.

**Methods:**

This prospective cross‐sectional study evaluated the concordance between SPT, ImmunoCAP, and ALEX‐2 in detecting respiratory allergen sensitization among 57 patients with asthma or AR recruited consecutively from a private allergy clinic in Lima, Peru. Concordance was assessed using Cohen's *κ* coefficient.

**Results:**

The highest overall agreement was observed between ALEX‐2 and SPT (*κ* = 0.5649, *p* < 0.0001), followed by ALEX‐2 and ImmunoCAP (*κ* = 0.4911, *p* < 0.0001), and ImmunoCAP and SPT (*κ* = 0.4688, *p* < 0.0001). ALEX‐2 and ImmunoCAP aligned on 7 of 10 allergens, while both ImmunoCAP and SPT, as well as ALEX‐2 and SPT, agreed on 5 of 9 allergens. The strongest concordance was found for dust mites, particularly *Dermatophagoides farinae* (*κ* = 0.6323, *p* < 0.0001) between ALEX‐2 and SPT.

**Conclusions:**

ALEX‐2 demonstrates moderate agreement with SPT and ImmunoCAP, with the highest concordance for dust mites, suggesting potential as a complementary diagnostic tool pending validation in diverse populations.

## 1 Introduction

Across Latin America, respiratory diseases like asthma and allergic rhinitis (AR) continue to challenge public health systems. International Study of Asthma and Allergies in Childhood (ISAAC) reveals *striking* patterns, with asthma prevalence reaching 33.1% among Peruvian children aged 6 to 7 years, a rate that underscores the urgency of addressing this health burden [1, 2], while allergic rhinitis affects children at rates ranging from 29.5% in Mexico to 49.9% in Brazil [3, 4, 5]. For clinicians working in this challenging environment, accurate identification of allergic sensitization becomes paramount.

The skin prick test (SPT) has long been the standard approach for allergic diagnosis, appreciated for its reliability, affordability, and patient tolerance [6]. In vitro methods like ImmunoCAP complement SPT by providing quantifiable specific IgE measurements across multiple allergens, particularly when SPT has limitations such as restricted allergen panels per session [7]. While earlier immunoblotting techniques once played a role in protein detection, ImmunoCAP has largely superseded these methods through its superior precision and efficiency in identifying allergen sensitization [8]. Despite these advantages, ImmunoCAP assesses allergens individually, requiring multiple tests for comprehensive evaluation.

ALEX‐2 (Allergy Explorer version 2), available since 2019, is a multiplex platform that detects multiple allergens simultaneously from a single sample, unlike traditional singleplex approaches like ImmunoCAP, which examine one allergen at a time [9]. This capability proves particularly valuable when evaluating patients with complex sensitization patterns that might elude conventional testing methods. The strategic combination of singleplex and multiplex testing approaches opens new possibilities for personalized treatment strategies, including targeted immunotherapy [10].

Although multiplex serological tests such as MAST (Multiple Allergen Simultaneous Test) have been available for decades, ALEX‐2 represents a more advanced microarray platform with expanded allergen coverage. Previous studies evaluating multiplex platforms have shown variable concordance with traditional methods; for instance, MAST‐immunoblot demonstrated moderate agreement with SPT (sensitivity 63%, correlation coefficients 0.27–0.36 for common allergens), highlighting the importance of validating newer platforms against established diagnostic methods. Such validation remains particularly needed in Latin American populations where allergen exposure patterns may differ from other regions [11, 12].

The technical capabilities of ALEX‐2 extend beyond simple multiplexing. The platform can measure specific IgE sensitization to over 295 allergens and molecular components simultaneously. This comprehensive profiling includes detailed molecular information that helps clinicians distinguish between clinically relevant and subclinical sensitization, thereby improving both risk assessment and management strategies for patients with intricate sensitization profiles [13].

While preliminary research has begun exploring the concordance between ALEX‐2, immunoblotting, and SPT with encouraging results for potential implementation in Peruvian clinical settings [14], a critical gap remains. The comparative performance of ALEX‐2, ImmunoCAP, and SPT has yet to be systematically evaluated in a single study. This represents the first exploratory investigation to examine the concordance among these three diagnostic modalities in detecting respiratory allergens among asthma and AR patients in Lima, Peru. Our findings aim to inform the design of larger, more comprehensive validation studies across diverse geographical and clinical contexts throughout Latin America.

## 2 Materials and Methods

### 2.1 Study Design and Patients

This prospective, observational, cross‐sectional study was designed as an observational, cross‐sectional study aimed at assessing the agreement between three methods for identifying allergic sensitization: ALEX‐2, ImmunoCAP and SPT. All three tests were performed on each patient with asthma or AR to enable direct comparison of results. The research took place in a private medical center in Lima, Peru, during the last quarter of 2024. The study focused on respiratory allergens such as dust mites, moulds, and animal dander.

Patients with clinically diagnosed asthma or AR confirmed by medical records were included if they provided informed consent (or parental consent for minors) and agreed to undergo all three diagnostic tests. Patients were excluded if they had severe chronic non‐allergic diseases that could confound results, were pregnant, were receiving ongoing allergen immunotherapy, or had insufficient blood sample volume.

To determine the sample size, Epidat Version 4.2 software was used, considering a 95% confidence level and a 5% precision. Following the reference study by Platteel et al [15]. An expected Cohen's *κ* value of 0.9 was established. Based on these parameters, a sample size of 61 patients was determined. These patients, who had a diagnosis of asthma or AR, were recruited consecutively as they presented at the clinic and met the inclusion criteria. However, due to insufficient blood samples for the ImmunoCAP test in four patients, the final sample size for analysis was reduced to 57.

### 2.2 Data Collection Techniques

The study collected demographic and clinical data via structured interviews, including allergy histories. AR diagnosis and severity classification were confirmed through medical records according to ARIA (Allergic Rhinitis and its Impact on Asthma) guidelines, categorizing patients as intermittent or persistent, and mild or moderate‐severe. Asthma diagnosis and severity were confirmed using GINA (Global Initiative for Asthma) criteria, classifying patients according to symptom patterns and disease control. Patients then underwent three sensitization tests performed during the same clinical visit to ensure temporal consistency. To minimize bias, all tests were performed by trained personnel using standardized procedures and protocols. SPT, performed by a trained nurse with prior experience in standardized allergy testing procedures from the immunology laboratory, involved applying allergenic extracts to the forearm with a small puncture to observe reactions. Blood samples were collected for ALEX‐2 and ImmunoCAP tests. For the serological assays, ImmunoCAP is a fluorescence enzyme immunoassay quantifying allergen‐specific IgE using purified allergens bound to a solid‐phase matrix [16]. ALEX‐2 is a multiplex microarray utilizing nano‐bead technology to simultaneously measure specific IgE against multiple allergen extracts and molecular components, with automated CCD blocking to reduce false positives [13, 14]. Analysis focused solely on respiratory allergen data from both platforms.

SPT positivity results were calculated by wheal diameter was ≥ 3 mm larger than the negative control, while the outcomes from ALEX‐2 and ImmunoCAP were expressed in kUA/L. ImmunoCAP was considered positive if the values were ≥ 0.35 kUA/L, and for ALEX‐2, if they were ≥ 0.30 kUA/L. It is important to note that the positivity thresholds applied for each test were established following the validated specifications provided by the respective manufacturers for each diagnostic platform, thus ensuring appropriate clinical interpretation according to the recommended standards for each technology. Concordance was calculated specifically for respiratory allergens. All information was systematically recorded in a Microsoft Excel database, with access restricted to the research team to ensure confidentiality.

### 2.3 Statistical Analysis

The analysis, conducted in STATA 18, assessed concordance among ALEX‐2, ImmunoCAP, and SPT based on sensitization presence or absence for nine shared allergens. An additional allergen, cockroach (Blattella germanica), was included for the ALEX‐2 and ImmunoCAP comparison, but excluded from the SPT due to differing species (Periplaneta americana in SPT). Cohen's kappa coefficient, along with positive and negative agreement, was calculated for each allergen. *κ* values were interpreted as follows: 0.01–0.20 slight, 0.21–0.40 fair, 0.41–0.60 moderate, 0.61–0.80 substantial, and 0.81–1.00 near‐perfect or perfect agreement.

## 3 Results

### 3.1 Demographic and Clinical Characteristics

Table 1 shows the demographic and clinical characteristics of participants. The median age was 22 years (range 4–58 years). Women represented 64.91% (*n* = 37) of the cohort, while men comprised 35.09% (*n* = 20). For rhinitis, the most common type was persistent moderate‐severe (40.35%, *n* = 23), followed by persistent mild (28.07%, *n* = 16) and intermittent moderate‐severe (22.81%, *n* = 13); intermittent mild rhinitis was the least common (7.02%, *n* = 4). Regarding asthma, persistent mild asthma was present in 17.54% (*n* = 10), while intermittent and persistent moderate asthma each affected 1.75% (*n* = 1).

**Table 1:** Demographic and clinical characteristics of patients with rhinitis and asthma.

Characteristic	Value
Age (years) Median (range)	22 (4–58)
Sex	
Women *n* (%)	37 (64.91%)
Men *n* (%)	20 (35.09%)
Rhinitis	
Intermittent mild *n* (%)	4 (7.02%)
Intermittent moderate‐severe *n* (%)	13 (22.81%)
Persistent mild *n* (%)	16 (28.07%)
Persistent moderate‐severe *n* (%)	23 (40.35%)
Asthma	
Intermittent asthma *n* (%)	1 (1.75%)
Persistent mild *n* (%)	10 (17.54%)
Persistent moderate *n* (%)	1 (1.75%)
IgE Total	
ImmunoCAP (kU/L) Median (range)	269 (8.8–19162)
ALEX‐2 (kU/L) Median (range)	233 (20–2500)


Abbreviations: IgE = Immunoglobulin E, kU/L = kilounits per liter.

Total IgE levels were assessed with ALEX‐2 and ImmunoCAP. ImmunoCAP showed a median total IgE of 269 kU/L (range 8.8–19,162 kU/L), and ALEX‐2 reported a median of 233 kU/L (range 20–2500 kU/L).

### 3.2 Agreement Between ImmunoCAP and SPT for Selected Allergens

Table 2 summarizes the agreement between ImmunoCAP and SPT for selected allergens, measured by the *κ* coefficient. The global *κ* coefficient for all allergens tested was 0.4688 (*p* = 0.0001), reflecting moderate overall agreement between ImmunoCAP and SPT across the selected allergens. The highest *κ* values were observed for *Dermatophagoides pteronyssinus* and *Blomia tropicalis*, with *κ* values of 0.4375 (*p* = 0.0007) and 0.4299 (*p* = 0.0007), respectively.

**Table 2:** *κ* coefficient and agreement between immunoCAP and Skin prick test for selected allergens.

N°	Allergen type	ImmunoCAP−SPT−	ImmunoCAP−SPT+	ImmunoCAP+SPT−	ImmunoCAP+SPT+	Agreement	Expected agreement	*κ*	*p* value
1	*Dermatophagoides pteronyssinus*	3	3	3	45	88.89	80.25	0.4375	0.0007
2	*Dermatophagoides farinae*	3	4	4	43	85.19	77.43	0.3435	0.0058
3	*Cladosporium herbarum*	50	4	0	0	92.59	92.59	0	—
4	*Aspergillus fumigatus*	51	2	1	0	94.44	94.58	−0.0253	0.5785
5	*Alternaria alternata*	52	2	0	0	96.3	96.3	0	—
6	Cat dander	34	4	10	6	74.07	62.83	0.3026	0.0099
7	Dog dander	26	3	14	11	68.52	51.78	0.3471	0.0024
8	*Blomia tropicalis*	7	4	7	36	79.63	64.27	0.4299	0.0007
9	*Penicillium notatum*	53	1	0	0	98.15	98.15	0	—
Total*	ImmunoCAP and SPT (Global)							0.4688	0.0001

*Note:* SPT = skin prick test; − negative result; + positive result. *κ* interpretation: 0.01–0.20 slight, 0.21–0.40 fair, 0.41–0.60 moderate, 0.61–0.80 substantial, 0.81–1.00 near‐perfect agreement.

* Global *κ* calculated across all allergens.

Fair agreement was found for Dog dander and *Dermatophagoides farinae*, with *κ* values of 0.3471 (*p* = 0.0024) and 0.3435 (*p* = 0.0058), respectively. Cat dander also showed fair agreement, with a Kappa value of 0.3026 (*p* = 0.0099). In contrast, moulds such as *Cladosporium herbarum*, *Alternaria alternata*, and *Penicillium notatum* had *κ* values of 0.00, indicating no agreement between the two methods for these allergens. *Aspergillus fumigatus* exhibited a negative *κ* value (−0.0253, *p* = 0.5785), also indicating no significant correlation.

### 3.3 Agreement Between ALEX‐2 and SPT for Selected Allergens

In the assessment of concordance between ALEX‐2 and the SPT, as shown in Table 3, varying levels of agreement were observed across different allergens. The overall Kappa coefficient across all allergens was 0.5649 (*p* < 0.0001), indicating moderate global agreement between the two methods for detecting allergic sensitization. The strongest concordance was found for Dermatophagoides farinae, with a substantial Kappa value of 0.6323 (*p* < 0.0001) and an agreement rate of 92.98%. Dermatophagoides pteronyssinus also exhibited moderate agreement, with a *κ* value of 0.4344 (*p* = 0.0004) and an agreement rate of 84.21%.

**Table 3:** *κ* coefficient and agreement between ALEX‐2 and skin prick test for selected allergens.

N°	Allergen type	ALEX‐2 − SPT−	ALEX‐2 − SPT+	ALEX‐2 + SPT−	ALEX‐2 + SPT+	Agreement	Expected agreement	*κ*	*p*‐value
1	*Dermatophagoides pteronyssinus*	5	6	3	43	84.21	72.08	0.4344	0.0004
2	*Dermatophagoides farinae*	4	0	4	49	92.98	80.92	0.6323	< 0.0001
3	*Cladosporium herbarum*	53	4	0	0	92.98	92.98	0	—
4	*Aspergillus fumigatus*	54	2	1	0	94.74	94.86	−0.024	0.5763
5	*Alternaria alternata*	55	2	0	0	96.49	96.49	0	—
6	Cat dander	34	2	12	9	75.44	58.08	0.4141	0.0003
7	Dog dander	35	6	7	9	77.19	60.39	0.4242	0.0007
8	*Blomia tropicalis*	11	3	6	27	66.67	53.19	0.288	0.0121
9	*Penicillium notatum*	56	1	0	0	98.25	98.25	0	—
Total	ALEX‐2 and SPT (Global)							0.5649	< 0.0001

*Note:* SPT = Skin Prick Test; − negative result; + positive result. *κ* interpretation: 0.01–0.20 slight, 0.21–0.40 fair, 0.41–0.60 moderate, 0.61–0.80 substantial, 0.81–1.00 near‐perfect agreement.

Fair agreement was observed for Cat dander (*κ* = 0.4141, *p* = 0.0003) and Dog dander (*κ* = 0.4242, *p* = 0.0007), with agreement rates of 75.44% and 77.19%, respectively. *Blomia tropicalis* showed a lower level of concordance, with a fair *κ* value of 0.288 (*p* = 0.0121) and an agreement rate of 66.67%.

Conversely, certain allergens such as *Cladosporium herbarum*, *Aspergillus fumigatus*, and *Penicillium notatum* showed little to no variability, resulting in *κ* values of 0 or negative, indicating no significant correlation between ALEX‐2 and SPT for these allergens.

### 3.4 Agreement Between ALEX‐2 and ImmunoCAP for Selected Allergens

Table 4 presents a comparison of the concordance between ALEX‐2 and ImmunoCAP for selected allergens. The global *κ* coefficient for agreement between these two methods, excluding the allergen *Blattella germanica*, was 0.4911 (*p* < 0.0001), indicating moderate overall agreement across the allergens tested.

**Table 4:** *κ* coefficient and agreement between ALEX‐2 and ImmunoCAP for selected allergens.

N°	Allergen Type	ALEX‐2−ImmunoCAP−	ALEX‐2−ImmunoCAP+	ALEX‐2+ImmunoCAP−	ALEX‐2+ImmunoCAP+	Agreement	Expected agreement	*κ*	*p* value
1	*Dermatophagoides pteronyssinus*	4	5	3	45	85.96	75.81	0.4198	0.007
2	*Dermatophagoides farinae*	5	10	3	39	77.19	67.04	0.3081	0.0061
3	*Cladosporium herbarum*	57	0	0	0	100	100	—	—
4	*Aspergillus fumigatus*	56	0	0	1	100	96.55	1	< 0.0001
5	*Alternaria alternata*	57	0	0	0	100	100	—	—
6	Cat dander	35	2	5	15	87.72	56.02	0.7208	< 0.0001
7	Dog dander	35	10	2	10	78.95	58.63	0.4911	< 0.0001
8	*Blomia tropicalis*	11	11	1	34	78.95	56.6	0.5149	< 0.0001
9	*Penicillium notatum*	57	0	0	0	100	100	—	—
10	Cockroach	42	11	1	3	78.95	71.87	0.2516	0.0075
Total*	ALEX‐2 and ImmunoCAP (Global)							0.4911	< 0.0001

*Note:* − negative result; + positive result. *κ* interpretation: 0.01–0.20 slight, 0.21–0.40 fair, 0.41–0.60 moderate, 0.61–0.80 substantial, 0.81–1.00 near‐perfect agreement. *Global *κ* calculated without *Blattella germanica* (cockroach).

Perfect agreement was observed for *Aspergillus fumigatus*, with a *κ* value of 1.0000 (*p* < 0.0001). Substantial agreement was found for *Cat dander* (*κ* = 0.7208, *p* < 0.0001), with an agreement rate of 87.72%. Moderate agreement was noted for *Blomia tropicalis* (*κ* = 0.5149, *p* < 0.0001), *Dog dander* (*κ* = 0.4911, *p* < 0.0001), and *Dermatophagoides pteronyssinus* (*κ* = 0.4198, *p* = 0.0070).

Fair agreement was identified for *Dermatophagoides farinae* (*κ* = 0.3081, *p* = 0.0061) and *Blattella germanica* (*κ* = 0.2516, *p* = 0.0075). Conversely, no variability was observed in the results for *Cladosporium herbarum*, *Alternaria alternata*, and *Penicillium notatum*, leading to undefined *κ* values.

## 4 Discussion

Our investigation into the concordance among SPT, ImmunoCAP, and ALEX‐2 for detecting respiratory allergens in Lima patients reveals interesting patterns. The strongest concordance was between ALEX‐2 and SPT (*κ* = 0.5649), despite these methods having different underlying principles: one relies on immediate skin reactions, the other on laboratory‐based molecular detection. ImmunoCAP and ALEX‐2 showed lower concordance (*κ* = 0.4911), despite both being laboratory methods, while ImmunoCAP and SPT had the lowest agreement (*κ* = 0.4688). These moderate agreement levels across all three methods reflect the complexity of assessing allergic sensitization. Each testing approach appears to capture different aspects of the allergic response, suggesting they are complementary rather than interchangeable. ALEX‐2 was the common denominator in both higher‐concordance pairings, indicating its role as a bridge between skin‐based and serum‐based testing. For clinicians in Peru and across Latin America, these findings offer diagnostic strategies that could improve both accuracy and efficiency in allergy evaluation.

When examining concordance patterns across methodologies, we observed varying agreement levels that must be interpreted within the context of each allergen panel. For the 9 allergens evaluated by all three methods, both the ImmunoCAP‐SPT and ALEX‐2‐SPT pairings demonstrated significant agreement for 5 allergens. When comparing ALEX‐2 and ImmunoCAP specifically, significant concordance was observed for 7 of 10 allergens, though this includes *Blattella germanica*, which was not assessed in the SPT panel. Regarding *Blattella germanica*, ALEX‐2 demonstrated capacity to detect specific molecular components Bla g 9 and Bla g 2, the latter being one of the major cockroach allergens [16], potentially contributing to the observed concordance patterns. The results underscore notable differences in method performance, especially with specific environmental allergens. Moderate agreement between ALEX‐2, ImmunoCAP, and SPT for *Dermatophagoides pteronyssinus* suggests reliability in detecting sensitization to this common allergen. For mould allergens, concordance varied: ALEX‐2 and ImmunoCAP showed perfect agreement for *Aspergillus fumigatus* (*κ* = 1.0, *p* < 0.0001), indicating effective sensitization detection. However, the lack of significant concordance for *Cladosporium herbarum* suggests possible limitations in detecting sensitization to certain mould species.

In some cases, calculating the *κ* coefficient was not possible due to a lack of variability in the results. For example, in the comparison between ALEX‐2 and ImmunoCAP for *Alternaria alternata*, all 57 samples were negative for both tests (ALEX‐2 − /ImmunoCAP − ), leading to perfect agreement but making it impossible to calculate *κ* due to the absence of positive results in either method. This lack of variability prevented the calculation of *κ*, as the coefficient requires both positive and negative results from both tests to assess concordance effectively. The variation in agreements can be attributed to the *κ* coefficient's sensitivity to the prevalence of positive and negative results. Even with high agreement percentages, an imbalance in outcome distribution can reduce the *κ* value and impact the detection of statistically significant agreements [17]. To address this limitation, we included both *κ* values and simple agreement percentages throughout our analysis to provide a more comprehensive interpretation of the concordance patterns.

The inconsistent agreement patterns observed across different allergens, particularly with moulds, may reflect Peru's geographical diversity. Lima's coastal desert environment differs substantially from other regions; for instance, Arequipa has documented notably higher mould sensitization rates [18], reflecting contrasting ecological conditions. Such regional variations in allergen exposure and environmental factors likely influence both sensitization patterns and diagnostic test performance, suggesting that region‐specific validation studies are needed for multiplex platforms like ImmunoCAP and ALEX‐2, particularly where certain allergens dominate or present unique molecular signatures [19].

Despite these geographical considerations and the variable performance we observed across allergen types, skin tests remain central to allergy diagnosis. Their simplicity, general safety profile, and reliability when conducted by trained personnel using standardized extracts continue to make them indispensable [20]. Yet there are times when serum IgE‐based methods become not just useful, but necessary, particularly when skin testing is contraindicated or when clinical circumstances make blood‐based alternatives more appropriate [21]. Our findings underscore something many practitioners intuitively understand: using multiple diagnostic methods provides a more complete picture of sensitization patterns, especially for common allergens like Dermatophagoides species. The challenge lies in balancing the comprehensive information gained from multiple testing approaches with practical realities like accessibility and cost, factors that ultimately determine whether these methods reach the patients who need them most.

When we examine the technical aspects more closely, ALEX‐2 and ImmunoCAP each have distinct advantages. ImmunoCAP has established itself as a standard tool in allergy laboratories worldwide, using automated quantitative immunoassays to measure specific IgE levels against whole allergen extracts with high standardization [22]. ALEX‐2 uses a different approach, employing microarray technology to simultaneously detect IgE against 295 allergens and molecular components, allowing for greater precision in molecular allergy assessment. Additionally, ALEX‐2 showed better concordance with SPT than methods like Immunoblot [14], a finding consistent with our observations and suggesting this technology may bridge the gap between traditional skin testing and molecular diagnostics. A study evaluating the first version of ALEX demonstrated substantial agreement among the three diagnostic approaches, particularly between ALEX and ImmunoCAP. ALEX showed significant concordance with both ImmunoCAP and SPT at a qualitative level, with a *κ* coefficient of 0.73 when comparing ImmunoCAP with SPT and 0.62 when comparing ALEX with SPT [23]. These findings are consistent with the results observed in the present study. In this study, ALEX‐2 and SPT showed similar agreement levels to a previous Peruvian study; however, while the prior study found significant agreement for *Alternaria alternata* [14], this study noted significance for dog dander. This difference may stem from unique environmental exposures in each city or from using specific molecular components like Can f 5, known for its high sensitization potential, to define dog dander positivity [24].

Important methodological considerations warrant careful attention when interpreting our findings. Although the sample size was calculated using standard epidemiological methods, four participants were excluded due to insufficient serum for ImmunoCAP testing, resulting in a final analytical sample of 57 participants. The study was conducted at a single private medical center in Lima, chosen specifically for its access to advanced allergen sensitization tools like ALEX‐2 and ImmunoCAP, which are not widely available elsewhere in Peru, thus limiting generalizability. Additionally, specific environmental factors unique to the urban setting of Lima were not systematically accounted for and may have influenced the concordance results across different allergen categories. Regarding statistical limitations, our analysis revealed instances of zero or negative *κ* values, particularly for mould allergens such as *Cladosporium herbarum*, *Alternaria alternata*, and *Penicillium notatum*. This statistical phenomenon reflects a well‐documented limitation of the kappa coefficient when examining allergens with extremely low prevalence in the study population. Despite observing high levels of agreement between tests (over 90% in some cases), we obtained *κ* values of zero or undefined results because *κ* is sensitive to imbalances between positive and negative outcomes. To address this limitation, we included both *κ* values and simple agreement percentages throughout our analysis to provide a more comprehensive interpretation of the concordance patterns. Finally, we intentionally chose not to establish any single test as a gold standard, as our aim was to evaluate all three diagnostic approaches as complementary methods. Designating one test as the reference standard would have fundamentally altered our methodological approach, creating an analytical framework where direct comparison between the two non‐reference methods becomes challenging and potentially limiting our ability to fully characterize the three‐way relationship we sought to examine.

The economic considerations prove particularly relevant in Peru's healthcare context. While SPT represents one of the more affordable options for allergen sensitization assessment, both SPT and serum IgE tests often come with out‐of‐pocket expenses that can burden families. Interestingly, research from Medicare beneficiaries revealed an unexpected finding: serum IgE testing, despite being more expensive upfront, was associated with lower overall costs and fewer specialist visits. This cost savings stemmed largely from practical advantages, such as not needing to discontinue antihistamines or requiring multiple appointments [25].

## 5 Conclusion

In summary, this exploratory study provides preliminary evidence of moderate agreement among ALEX‐2, ImmunoCAP, and SPT in identifying respiratory allergen sensitization. ALEX‐2 and ImmunoCAP agreed on 7 of 10 allergens, while ImmunoCAP and SPT, as well as ALEX‐2 and SPT, agreed on 5 of 9 allergens. Our findings suggest that these methods are complementary rather than interchangeable, with each capturing different aspects of the allergic response. ALEX‐2 showed moderate concordance with established methods, though its utility varies by allergen type. Diagnostic method selection should consider the clinical context and available resources. Future multicenter studies with larger, more diverse populations and standardized allergen panels are essential to validate these preliminary findings and determine the appropriate clinical role of multiplex platforms in Latin American healthcare setting.

## Author Contributions

**César A. Galván:** conceptualization, methodology, project administration, resources, writing – original draft, writing – review and editing. **Rafael Durán:** data curation, formal analysis, investigation, methodology, software, writing – original draft. **José Ignacio Larco:** formal analysis, writing – original draft, writing – review and editing. **Juan Carlos Gomez de la Torre:** methodology, writing – review and editing.

## Funding

The authors received no specific funding for this work.

## Ethics Statement

The Ethics Committee of Hospital San Bartolomé granted ethical approval for this study (protocol number 10268‐24). All procedures followed the ethical standards established by the institutional research committee and adhered to the principles outlined in the Declaration of Helsinki.

## Consent

All study participants provided informed consent before enrollment. For participants under 18 years of age, parents or legal guardians provided informed consent on their behalf, while the minors themselves gave written informed assent following established ethical guidelines. The research team maintained strict confidentiality of all participant data throughout the entire study period.

## Conflicts of Interest

César A. Galván has given educational talks for Pharmedic International (ALEX‐2 distributor in Peru). Other authors declare no conflicts of interest.

## Data Availability

The data that support the findings of this study are available from the corresponding author upon reasonable request.
